# Sensitivity-Enhanced Extrinsic Fabry–Perot Interferometric Fiber-Optic Microcavity Strain Sensor

**DOI:** 10.3390/s19194097

**Published:** 2019-09-22

**Authors:** Zhibo Ma, Shaolei Cheng, Wanying Kou, Haibin Chen, Wei Wang, Xiongxing Zhang, Tongxin Guo

**Affiliations:** 1 Shaanxi Key Lab of MEMS/NEMS, Northwestern Polytechnical University, Xi’an 710072, China; zbma@nwpu.edu.cn (Z.M.); cheng_sl@mail.nwpu.edu.cn (S.C.); guo-tx9199@mail.nwpu.edu.cn (T.G.); 2 Key Lab of Micro/Nano Systems for Aerospace, Ministry of Education, Northwestern Polytechnical University, Xi’an 710072, China; 3 Xi’an Technological University, School of Optoelectronics Engineering, Xi’an 710021, China; kouwyxatu@163.com (W.K.); chenhaibin@xatu.edu.cn (H.C.); zhangxiongxing@xatu.edu.cn (X.Z.); 4 Shaanxi Province Key Lab of Photoelectric Measurement and Instrument Technology, Xi’an Technological University, Xi’an 710021, China

**Keywords:** strain sensor, fiber-optic sensor, Fabry–Perot cavity, sensitivity

## Abstract

This study presents an extrinsic Fabry–Perot interferometric (EFPI) fiber-optic strain sensor with a very short cavity. The sensor consists of two vertically cut standard single-mode fibers (SMFs) and a glass capillary with a length of several centimeters. The two SMFs penetrate into the glass capillary and are fixed at its two ends with the use of ultraviolet (UV) curable adhesives. Based on the use of the lengthy glass capillary sensitive element, the strain sensitivity can be greatly enhanced. Experiments showed that the microcavity EPFI strain sensor with initial cavity lengths of 20 μm, 30 μm, and 40 μm, and a capillary length of 40 mm, can yield respective cavity length–strain sensitivities of 15.928 nm/με, 25.281 nm/με, and 40.178 nm/με, while its linearity was very close to unity for strain measurements spanning a range in excess of 3500 με. Furthermore, the strain–temperature cross-sensitivity was extremely low.

## 1. Introduction

Strain is one of the most important parameters that needs to be continually measured for monitoring buildings, bridges, railways, tunnels, and marine and aerospace engineering structures [1,2,3,4,5,6,7,8]. In view of their advantages of compact size, high sensitivity, immunity to electromagnetic interference, chemical passivity, and broad operating range, fiber-optic strain sensors have attracted attention in recent years the academia and industry.

Several types of fiber-optic strain sensors have been proposed. Most of them are based on fiber Bragg gratings (FBGs) [9,10,11], long period fiber gratings (LPFGs) [12,13], in-fiber Mach–Zehnder (MZ) interferometers [14,15,16,17], and Fabry–Perot (FP) interferometers [18,19,20]. The FBG strain sensors use the linear response of the Bragg wavelength of the FBG to conduct the strain measurements. Specifically, through wavelength multiplexing, multipoint strain monitoring can be easily realized. In the case of the LPFG strain sensors, when a broadband light is illuminated on the sensor, some resonant peaks will appear in the transmission spectrum, which can be linearly shifted by the imposed strain. The MZ interferometric strain sensors can split the incident light into two or more different modes, such as the core and cladding modes in a single mode fiber, the two (or more) guiding modes in a multimode, two-core, or multicore fibers. Given that the modes are associated with different optical paths, and given that the optical path difference can be effectively varied by the imposed strain, the interfering peaks in the transmission spectrum have a definite relationship with the strain that can be used to extract the strain information.

However, all these types of fiber-optic strain sensors exhibit some temperature sensitivity, while the cross sensitivity between the strain and the temperature restricts their real applications. Some well-conceived methods have been proposed [21,22,23] to eliminate the cross influence of temperature on the strain. However, the methods that unavoidably led to increased process complexity in the case of the sensing system and fabrication process also led to increased costs.

The inline FP interferometric strain sensors can be realized in many different ways. These types of sensors can be classified into two types: the intrinsic FP interferometric (IFPI) strain sensors [24], and the extrinsic FP interferometric (EFPI) strain sensors [25,26,27,28,29,30]. The EFPI strain sensors have lower sensitivities to temperature and are insensitive to transverse strain. Accordingly, they have traditionally received more attention. In the case of the EFPI strain sensor, an FP air-cavity is introduced into the fiber to achieve the strain measurement through the FP interfering effect. An EFPI strain sensor can be fabricated by splicing a short silica capillary [25] or a hollow-core photonic crystal fiber [26] between two standard fibers. The EFPI strain sensing can also be achieved by the spherical [27], elliptical [28,29], or rectangular [30] micro-air bubbles generated on the axis of an optical fiber. Compared to FBG, LPFG, and MZ interferometric strain sensors, EFPI strain sensors are more easily fabricated, and most importantly, they exhibit far less strain–temperature cross-sensitivity.

Both experiments and theoretical analyses have shown that the shorter cavity length allows the achieving of a higher strain sensitivity [31,32]. The *m*-th order reflection peak and the cavity length of an EFPI strain sensor has a relationship of $2L=m\lambda_{m}$, in which, *L* is the FP cavity length, $\lambda_{m}$ is the wavelength of the *m*-th order reflection or transmission peak, and *m* is an integer. For a short FP cavity length, the reflection peaks can be appeared in the reflection or transmission spectrum will have a relatively low order, i.e., *m* becomes a smaller number. For a cavity length variation ΔL, the wavelength variation is $\Delta \lambda_{m}=2\Delta L/m$. For a shorter cavity length, with the same cavity length variation, the wavelength variation will have a larger value. That is why when using a wavelength tracking method or similar method to extract the strain, a shorter cavity length of FP strain sensor will have a higher strain sensitivity. Thus, many researchers focused on the fabrication of microcavity FP strain sensors. Although many different types of EFPI microcavity strain sensors have been proposed, limited by the wavelength range of the light source can be used, and the fabrication difficulties, the cavity length cannot be infinitely reduced. As a result, the sensitivity increasing effect by the using of shorter cavity length is very limited. Typically, the wavelength–strain sensitivities spanned several pm/µε.

To further increase the strain sensitivity, several different methods have been proposed. Liu et al. made an in-fiber micro-air bubble strain sensor and reduced its sidewall thickness through a tapering process. Since a thinner sidewall can stretch a greater extend under the same stress, the cavity length-strain sensitivity was increased to a level of 43.0 pm/με [30]. However, the method sacrifices the dynamic range and mechanical strength. The strain sensitivity can also be amplified by the using of two cascaded FP cavities through the Vernier effect. Tian et al. reported the achieving of a wavelength-strain sensitivity of 1.15 nm/με through this method [33]. The main difficulty of this method is the optical lengths of the main sensing FP cavity and the matched FP cavity need to be precisely matched by carefully controlling the two cavity lengths.

Another effective way to increase the strain sensitivity is separate the strain active length from the FP cavity length. By increasing the ratio of the strain active length and the FP cavity length, the strain sensitivity can be effectively increased. Zhou et al. reported a type of all-fiber pillar-in-bubble FP strain sensor fabricated by a CO_2_ laser machining technique; the sensor got a highest wavelength–strain sensitivity of 56.69 pm/με when the ratio of active length against cavity length was 10 [34]. Pevec and Donlagic proposed an all fiber long active length FP strain sensor based on the introduction of a deep gutter into the cavity structure to increase the active length through a micromachining process [35]. The ratio of the strain active length and the FP cavity length was ~70.6, and the cavity length–strain sensitivity reached a value of 0.75 nm/με. Liu et al. reported an EFPI microcavity strain force sensor with ultrahigh sensitivity, which has a similar structure. They fabricated the sensor by plugging and fusing a cantilever taper into a hollow tube fused at the facet of a single mode fiber (SMF), the strain force sensor with a microcavity length of 3.5 μm and an active length of 1360 μm [36]. Obviously, one of the key issues to increase the strain sensitivity is to increase the ratio of the strain active length and the cavity length. However, the strain active length achieved by the micromachining process was very limited, which restricted further enhancement of the strain sensitivity.

In this study, a fiber-optic structure with an EFPI microcavity was proposed for strain sensing. It is simply formed by two SMFs which are separated by tens of micrometers and fixed in a centimeters-long glass capillary at its two ends. The proposed EFPI microcavity fiber-optic strain sensor has a considerably enhanced sensitivity within a large measurement range, since sensitivity increasing effects of the microcavity and the separation of the strain active length from the FP cavity length are combined. First, using the microcavity, the reflection spectrum becomes relatively sensitive to cavity length change under the influence of strain or stress, so, the cavity length change can be more accurately determined. Second, by the usage of the relatively long strain-sensitive, glass capillary, the ratio of the strain active length and the FP cavity length in principle is unlimited, the cavity length–strain sensitivity can be greatly enhanced.

## 2. Structure and Fabrication

**Figure 1:**
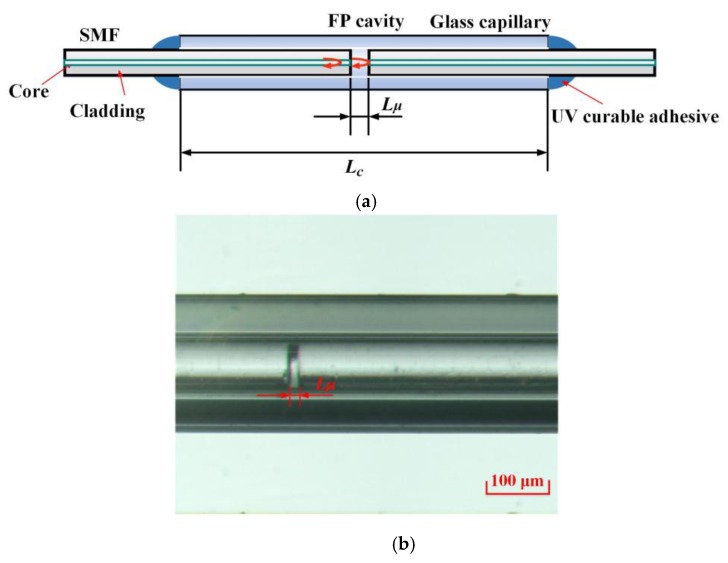
Structure of the extrinsic Fabry–Perot interferometric (EFPI) microcavity fiber-optic strain sensor. The sensor is composed of two vertically cut single mode fibers with a short facet separation in the level of micrometers and a centimeters-long glass capillary. (**a**) Schematic of the sensor, (**b**) microscopic photograph of the microcavity. In which, *L_μ_* and *L_C_* are the microcavity length and the capillary length, respectively.

The typical structure of the EFPI microcavity fiber-optic strain sensor consists of two SMFs and a glass capillary, as shown in Figure 1a. The two facets of the SMFs are parallel to each other, vertical to the central axis of the SMFs, and have a very short separation of the order of tens of micrometers. An EFPI microcavity is formed by the two facets of the SMFs. To fix the separation of the two SMFs to form a stable EFPI microcavity, a glass capillary with a length of a few centimeters was used. The two SMFs were fixed at the two ends of the glass capillary by UV-curable adhesives.

The fabrication procedure of the EFPI microcavity strain sensor included the following steps: (a) firstly, remove a short length of the coating layers at one end of the two SMFs, then vertically cut the SMFs to obtain two smooth and clean end-faces; (b) secondly, allow the two SMFs to penetrate into the glass capillary, which have a length of several centimeters, using a pair of translation stages with microscopic monitoring; (c) thirdly, connect the other end of one SMF with an optical spectrum analyzer (OSA) and a wideband light source, such as a superluminescent diode (SLD) or an amplified spontaneous emission (ASE) through a three-port optical circulator with the aid of the reflection spectrum to calculate the facet separation of the two SMFs (FP cavity length) in the glass capillary, and finely tune the two translation stages to achieve a cavity length that matches the predetermined value (tens of micrometers); (d) finally, place some UV-curable adhesives at the two ends of the glass capillary, use a UV lamp to illuminate the two ends, and fix the structure to obtain the EFPI microcavity fiber-optic strain sensor. The microscopic photograph of the fabricated EFPI microcavity fiber-optic strain sensor with a cavity length of 20 µm is shown in Figure 1b. It should be noted that the SMFs are only fixed at the positions of the two ends of the glass capillary, while the other parts have no mechanical connection with the glass capillary.

## 3. Measurement Principle and Sensitivity Enhancement

**Figure 2:**
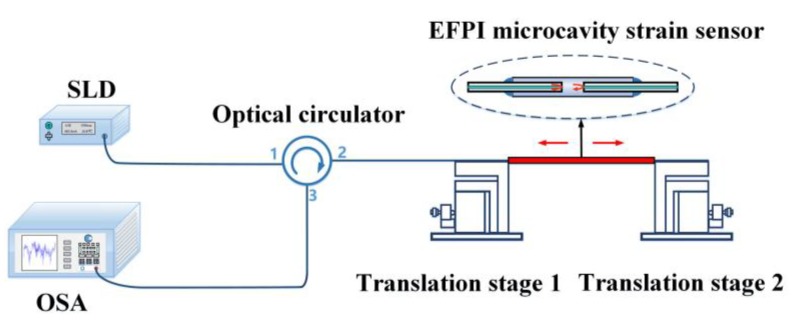
Schematic of a strain sensing system based on the EFPI microcavity strain sensor (SLD: superluminescent diode, OSA: optical spectrum analyzer). The SLD is used as a wideband illuminating source. The OSA is used to extract the reflection spectrum. Two capillary ends of the EFPI microcavity strain sensor are fixed on a pair of translation stages for strain imposing test.

A strain sensing system was used to conduct the strain measurements. As shown in Figure 2, this system consists of an SLD, an optical circulator, an OSA, and an EFPI microcavity strain sensor. A wideband light output from the SLD is irradiated into the EFPI microcavity strain sensor through a three-port optical circulator. In view of the interfering effect between the two facets of the microcavity in the strain sensor, the reflected light will be modulated and will contain the cavity length information of the EFPI microcavity strain sensor. The reflection light will return back to port 2 of the optical circulator, and the output from port 3 will be finally received by an OSA to obtain the reflection spectrum.

It should be noted that the distal SMF end of the EFPI microcavity strain sensor may also reflect some light if the facet is not specially treated to eliminate the reflection. There are many ways to eliminate the reflection of the distal end, such as roughing the surface through a chemical or mechanical way, coating it with an anti-reflection film, or cut the fiber with a tilted angle of 8°. If the reflection is not effectively reduced, the whole structure can be viewed as a compound FP cavity, or two cascaded FP cavities: one cavity formed by the air, which has a cavity length of 10–200 µm; and one cavity formed by the two reflecting surfaces of the out SMF, which has a cavity length of several centimeters. The reflection spectrum from the structure will be in a form of a fast oscillation (determined by the length and refractive index of the out SMF) modulated by a slowing varying envelope (determined by the length of the air microcavity). However, if the length of the out SMF is longer than 5 cm, the peak separation of the fast oscillation will be less than 0.018 nm, which can be automatically filtered by the OSA used in most cases. Thus, we can just keep the out SMF not too short and no special treatment is needed.

The two end-faces of the microcavity are the same. If we assume that they have the same reflection ratio R, for a light with a wavelength λ, the reflectivity $R_{FP}(\lambda )$ of the EFPI microcavity fiber-optic strain sensor can be expressed as

(1) $$R_{FP}\left(\lambda \right)=\frac{2R\left(1-\cos\frac{4\pi L_{\mu }}{\lambda }\right)}{1+R^{2}-2R\cos\frac{4\pi L_{\mu }}{\lambda }}$$

where $L_{\mu }$ is the cavity length of the FP microcavity.

The refractive index of the SMF is approximately 1.46 at the end-face with air as the surrounding medium, R≈0.035. Thus, Equation (1) can be approximately rewritten as

(2) $$R_{FP}\left(\lambda \right)\approx 2R\left(1-\cos\frac{4\pi L_{\mu }}{\lambda }\right).$$

**Figure 3:**
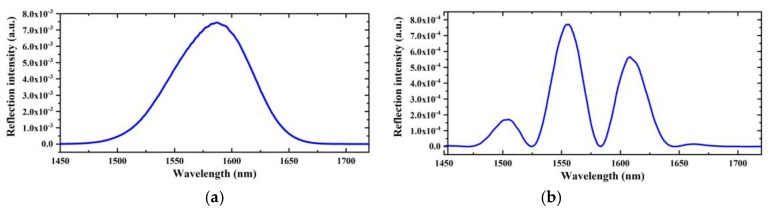
(**a**) Spectrum of a SLD with a center wavelength of 1568 nm and a 3 dB bandwidth of 98 nm, which has a Gaussian-like spectral distribution. (**b**) Reflection spectrum of an EFPI microcavity fiber-optic strain sensor, which exhibit several peaks can be used to determine the cavity length.

For wideband light illuminated from a SLD (similar to that shown in Figure 3a), the reflection spectrum of the EFPI microcavity fiber-optic strain sensor will be modulated and will exhibit several peaks, as seen in Figure 3b. Peak numbers, positions, and separations are determined by the cavity length.

For a reflection peak with an order of m, the wavelength position should satisfy

(3) $$\frac{4\pi L_{\mu }}{\lambda_{m}}=\left(2m+1\right)\pi ,m=0,1,2,\ldots$$

which means that

(4) $$4L_{\mu }=\left(2m+1\right)\lambda_{m}.$$

For another reflection peak with an order of q, the wavelength position should satisfy

(5) $$4L_{\mu }=\left(2q+1\right)\lambda_{q}.$$

From Equations (4) and (5), we have

(6) $$\frac{1}{\lambda_{m}}-\frac{1}{\lambda_{q}}=\frac{m-q}{2L_{\mu }}.$$

If both of the two reflection peaks can be found in the reflection spectrum, the cavity length can then be calculated by

(7) $$L_{\mu }=\frac{m-q}{2}\frac{\lambda_{m}\lambda_{q}}{\lambda_{q}-\lambda_{m}}.$$

Embedding of the EFPI microcavity in an object, or fixing it on its surface, allows the EFPI microcavity fiber-optic strain sensor to be deformed in unison with the object. When a stress is imposed on the strain sensor, the length of the cavity will be changed with the elongation of the entire structure. The microcavity length $L_{\mu }$ and the microcavity length variation $\Delta L_{\mu }$ can be extracted from the reflection spectrum of the EFPI. This is attributed to the fact that the entire glass capillary participates in the elongation, and the SMFs are fixed at the two ends of the glass capillary. The change of the cavity length is equal to the change of the length of the glass capillary. The strain value ε can be obtained directly and simply by

(8) $$\varepsilon =\frac{\Delta L_{\mu }}{L_{C}}$$

where $L_{C}$ is the length of the glass capillary.

The FP microcavity fiber-optic strain sensor fabricated by femtosecond laser writing or with direct splicing, commonly has an active strain length equal to *L_μ_*. When a strain ε is added, $\Delta {L^{\prime }}_{\mu }=\varepsilon L_{C}$. Obviously, $\Delta L_{\mu }/\Delta {L^{\prime }}_{\mu }=L_{C}/L_{\mu }$. It can be noted that at the same strain, the length elongation of the proposed FP microcavity fiber-optic strain sensor used in this study is much larger than others. The microcavity length is commonly extracted from the reflection spectrum on an OSA, and a larger change in the range of the cavity length will result in more obvious changes in the reflection spectrum. Additionally, the cavity length change can be extracted more easily to obtain the imposed strain. Thus, the strain sensitivity will be greatly enhanced.

## 4. Experiments and Data Analyses

**Figure 4:**
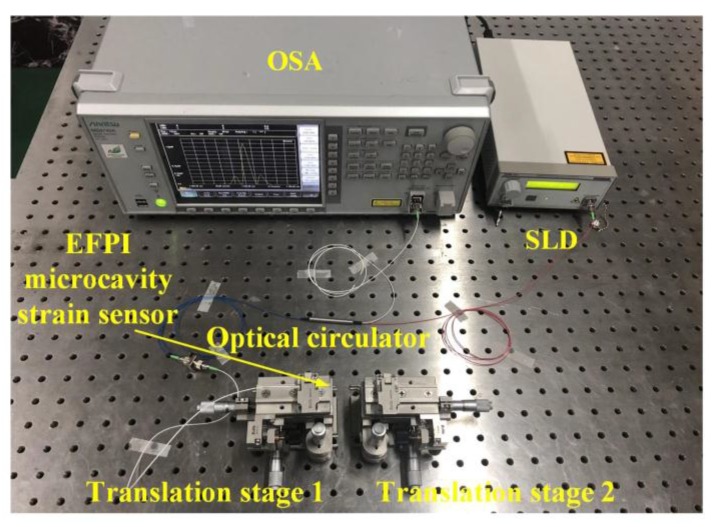
Photograph of the experimental system with the EFPI microcavity fiber-optic strain sensor. Two capillary ends of the sensor were fixed on the pair of translation stages through 502 glue. Axial direction moving of the translation stages is used to impose strain on the glass capillary of the sensor.

To characterize the strain sensing performances of the EFPI microcavity fiber-optic strain sensor, an experimental setup was built, as shown in Figure 4. The SLD (S5FC1550S–A2, Thorlabs Inc., Newton, NJ, USA) used has a center wavelength of 1568 nm and a 3 dB bandwidth of 98 nm. The spectrum of the SLD is shown in Figure 3a. The OSA used is a high-resolution fiber-optic type, with a spectral range of 0.6–1.75 μm, and a minimum wavelength resolution of ~30 pm.

For the fabrication of the EFPI microcavity fiber-optic strain sensor, standard SMFs (model G652D, Corning Inc., Corning, NY, USA) were used with core and cladding diameters of 9 µm and 125 µm, respectively. The glass capillary had an inner diameter of 128 μm and an outer diameter of 320 μm. We fabricated three EFPI microcavity strain sensors with the same capillary length of 40 mm with different FP cavity lengths (20 μm, 30 μm, and 40 μm).

**Figure 5:**
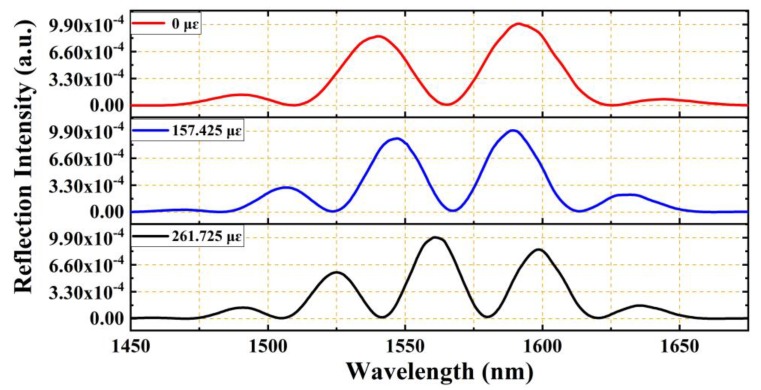
Spectral shift upon application of a longitudinal strain to a short cavity EFPI strain sensor with a microcavity length 20 µm of and a capillary length of 40 mm. The three reflection spectra were gotten under different strains of 0, 157.425, and 261.725 με, respectively. With the increasing of the strain imposed, more reflection peaks appeared in the spectra for the increasing of cavity length.

The strain sensors were fixed on a pair of translation stages, and the center of the EFPI microcavity was placed at the center position between the two stages. To prevent significant stress on the UV-curable adhesives used in the strain sensor at the two ends of the glass capillary, the glass capillary was directly adhered to the two translation stages by the using of 502 glues. The using of glues instead of mechanical clamping can prevent non-uniform stress being imposed on the sidewall of the glass capillary. Motion along the axial direction with the use of one translation stage led to the elongation of the glass capillary of the EFPI microcavity fiber-optic strain sensor, as shown in Figure 5. Accordingly, the interfering peaks on the reflection spectrum were red-shifted to the longer wavelength direction, and the wavelength separation between any two neighboring interfering peaks were continually and obviously decreased, as shown in Figure 5. The results indicate that the cavity length of the EFPI microcavity is very sensitive to the imposed strain.

**Figure 6:**
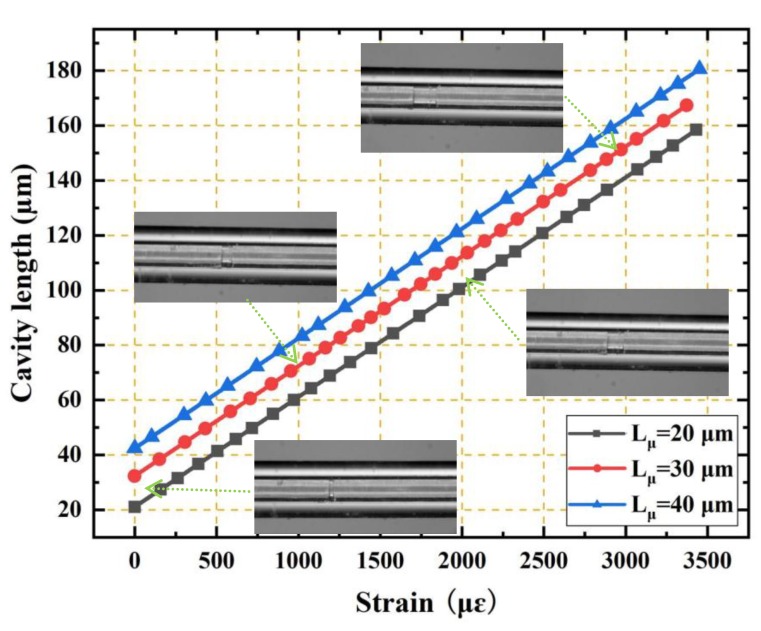
Relationships between the cavity length and the strain for three EFPI microcavity fiber-optic strain sensors with the same capillary lengths of 40 mm but different initial cavity lengths (20 μm, 30 μm, and 40 μm). The cavity length–strain sensitivity was ~40.459 nm/με, regardless of the initial cavity length. Micrographs of the EFPI microcavity fiber-optic strain sensor with the initial cavity length of 30 μm under four different strains of 0, 1000, 2000 and 3000 με are also given as insets.

The cavity length of the EFPI fiber-optic strain sensor can be calculated using Equation (6), and the relation between the cavity length and the strain can be obtained directly. Figure 6 shows the relationships between the cavity length and the strain imposed for the three EFPI microcavity fiber-optic strain sensors with the same capillary lengths of 40 cm and at different initial cavity lengths. It can be observed that the relationship between the cavity length and strain is linear with a slope that is very close to unity. The cavity length–strain sensitivity was ~40.459 nm/με, regardless of the initial cavity length. Additionally, the measured strains values were >3500 με.

**Figure 7:**
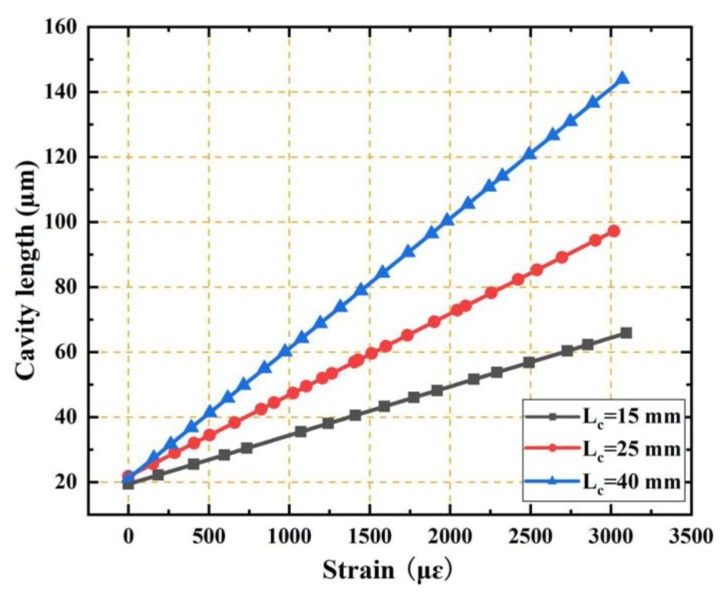
Relationships between the cavity length and the strain for three EFPI microcavity fiber-optic strain sensors with the same initial cavity length of 20 μm, but different capillary lengths (15 mm, 25 mm, and 40 mm). The cavity length–strain sensitivities of the three sensors were 15.928 nm/με, 25.281 nm/με, and 40.178 nm/με, respectively. Evidently, the sensitivity is directly proportional to the capillary length, and can be greatly enhanced by the capillary length.

We also investigated the influence of the capillary length on the strain sensing performance. Figure 7 shows the relationships between the cavity length and the strain for the three EFPI microcavity fiber-optic strain sensors with the same initial cavity length of 20 μm but at different capillary lengths of 15 mm, 25 mm, and 40 mm. The cavity length–strain sensitivities of the three sensors were 15.928 nm/με, 25.281 nm/με, and 40.178 nm/με, respectively. As shown, the sensitivity is directly proportional to the capillary length. The strain sensitivity can be greatly enhanced by the capillary length, and it can be tuned to the desired level by the selection of the capillary length.

Although the strain sensitivity can be greatly enhanced by the using of a longer capillary, it sacrifices the locality of the strain measurement to some extent. The longer the capillary length, the more nonlocal the strain measurement. This is the major drawback of the EFPI microcavity fiber-optic strain sensor. However, considering the enhancement extent of the strains sensitivity is directly determined by the ratio of the glass capillary length and the microcavity length $L_{C}/L_{\mu }$, we can reduce both lengths of the glass capillary and microcavity simultaneously to achieve strain measurement in a relatively small scale but that still maintains the strain sensitivity enhancement.

**Figure 8:**
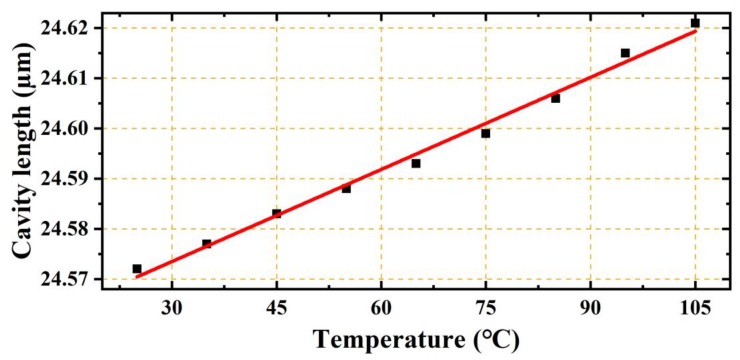
Relationship between the cavity length and the temperature for an EPPI microcavity fiber-optic strain sensor with a capillary length of 40 mm and an initial cavity length of 25 μm. The cavity length increased as a function of temperature, and the cavity length–temperature sensitivity was ~0.612 nm/°C.

The temperature response of the EFPI fiber-optic strain sensor was also investigated. The EFPI fiber-optic strain sensor with the capillary length of 40 mm, and the cavity length of 25 μm was inserted in a muffle furnace, and the temperature was adjusted in a range of 25–105 °C. As shown in Figure 8, the cavity length increased as a function of temperature, and the cavity length–temperature sensitivity was ~0.612 nm/°C. Even though this is relatively high, it is reasonable owing to the fact that the glass capillaries with lengths of the order of centimeters were all affected by the temperature change. However, the corresponding strain–temperature cross sensitivity was only approximately 0.0152 με/°C, which is extremely low.

## 5. Conclusions

In this study, a fiber-optic strain sensor was proposed based on an EPPI microcavity structure. By fixing two SMFs at each of the two ends of glass capillaries with lengths of the order of a few centimeters, short-length FP micro-cavities were formed that allowed the strain sensor to yield very high sensitivities for strain measurements. Three EPPI microcavity fiber-optic strain sensors were implemented with the same capillary length of 40 mm but with different FP cavity lengths. The highest cavity length–strain sensitivity was 40.459 nm/με. An extremely low strain–temperature cross sensitivity of 0.0152 με/°C was achieved, while the linearity was very close to unity within a strain measurement range of 3500 με. It can be concluded that the EPPI microcavity strain sensor has low-cost advantages, high sensitivity, a large measurement range, and increased linearity.
